# Smartphone usage in the 21st century: who is active on WhatsApp?

**DOI:** 10.1186/s13104-015-1280-z

**Published:** 2015-08-04

**Authors:** Christian Montag, Konrad Błaszkiewicz, Rayna Sariyska, Bernd Lachmann, Ionut Andone, Boris Trendafilov, Mark Eibes, Alexander Markowetz

**Affiliations:** Institute of Psychology and Education, Ulm University, Ulm, Germany; Department of Informatics, University of Bonn, Bonn, Germany; Molecular Psychology, Zentrum für Biomedizinische Forschung, Helmholtzstr. 8/1, 89081 Ulm, Germany

**Keywords:** Whatsapp, Personality, Extraversion, Conscientiousness, Age, Gender, Education

## Abstract

**Background:**

Mounting evidence shows that smartphone usage heavily disrupts our work life and social activities. Moreover, it is possible that overuse could resemble addictive tendencies. A key contributing factor to smartphone overuse seems to be usage of the messaging application WhatsApp. Although WhatsApp is one of the most commonly used communication applications on smartphones, research in this area is scarce. Given the huge societal debate on the impact of smartphone usage on our daily lives, the present study undertook a large-scale investigation in order to provide numbers on smartphone usage generally—and use of WhatsApp in particular, with the aim of providing a basis for a scientific debate.

**Methods:**

In a large sample of N = 2,418 users, we recorded WhatsApp behaviour over a 4 week period.

**Results:**

Our data show that use of WhatsApp accounted for 19.83% (= 32.11 min) of all smartphone behaviour (compare: Facebook only 9.38% = 15.19 min). The mean of general daily smartphone usage was 161.95 min. Females used WhatsApp for significantly longer periods of time than males and younger age was associated with longer duration of WhatsApp use. While the personality trait Extraversion was positively associated with daily WhatsApp use, Conscientiousness showed an inverse correlation with the length of daily WhatsApp use.

**Conclusions:**

The numbers on smartphone usage in the present study show that the smartphone dominates our daily life. In particular WhatsApp is a driving force, here. Given the length of daily smartphone and WhatsApp usage, more studies need to be conducted to better understand smartphone usage.

**Electronic supplementary material:**

The online version of this article (doi:10.1186/s13104-015-1280-z) contains supplementary material, which is available to authorized users.

## Background

Smartphones represent an important part of modern life, because they enable us to communicate from nearly everywhere (as long a phone signal is available), access the Internet, check e-mails and social networks. In Germany alone, 40% of the population use a smartphone [1] and overuse is heavily discussed in terms of a potential negative impact on mental health. It has been suggested that overuse of smartphones may resemble an addiction [2, 3] and overuse is also linked to time distortion, with the result that the smartphone is used for longer than originally intended or perceived [4]. Moreover, smartphone overuse (in particular evening usage) seems to be associated with poor sleep quality [5] and reduced work engagement [6], which is not surprising, as new numbers show that about 36–40% of smartphone owners use their smartphone in the last 5 min before going to sleep and/or in the first 5 min after waking up [7]. Thus, this technical device is omnipresent in everyday life. Most studies in this field rely on questionnaires into assess smartphone consumption. This is problematic, because a recent study showed that usage is poorly assessed via self-report questionnaires [4]. The aim of the present study is to provide numbers on smartphone usage and one major smartphone utility—WhatsApp—in order to provide an empirical basis for scientific discussion. Usage is further investigated in the context of personality.

WhatsApp is a communication app facilitating the exchange of instant messages, pictures, videos and voice calls via an Internet connection, which has been installed on smartphones over half a billion times all round the world [8].^a^ It represents one of the most important features of a smartphone, as it enables easy communication via text or voice messages between two or more persons. Basically it helps people to stay connected. WhatsApp is a particularly attractive as, after installing the app, the sending and receipt of messages is cost free (in contrast to the original text message function on mobile phones). The latter feature (cost free) clearly explains the success of WhatsApp. In addition, its function across different smartphone types (Apple, Android, etc.) and its international functionality are also important contributors to this popularity.

Scientific studies on WhatsApp usage remain scarce. Recent studies yielded the first evidence that WhatsApp usage might have an addictive character [9] and of course WhatsApp represents an important communication alternative to classic short, which often incur costs (SMS; [10]). In contrast to the small body of literature on WhatsApp, the past few years reflect a focus on social network usage, such as Facebook, when investigating smartphone behaviour or Internet usage (e.g. [11]). Moreover, it is noteworthy that most previous studies have relied solely on self-report data to measure human–machine interaction [12, 13]. The well-known shortcomings of self-report data, such as social desirability in answering questionnaires, can in part be overcome through a new research avenue called Psychoinformatics. Psychoinformatics adapts methods from computer science to the study of human behaviour, such as the interaction of a person with his/her smartphone [14, 15]. Crucially, behaviour is recorded directly. In this context, a study by our own group demonstrated that extraverted humans reach out to their social network more via voice calls. In particular, the number of outgoing calls was positively associated with Extraversion. Extraverts can be described as socially outgoing and attaching easily to other persons. Our pilot study underlined the importance of earlier findings by Chittaranjan et al. [16], who, to our knowledge, published one of the first studies on the link between personality and actual recorded smartphone behaviour. As the pilot study focused on call and SMS variables in a rather small sample, the purpose of the present investigation is to contribute to a better understanding of WhatsApp usage in a large number of users, tracked over a period of 4 weeks.

Specifically, the present study aims to answer three questions. First, we are interested to learn what percentage of all smartphone usage is specific to WhatsApp (although daily general usage is also of interest in this investigation). Second, we investigate how the socio-demographic variables age, gender and education influence WhatsApp usage. Finally, we are interested in linking personality to WhatsApp usage. In light of earlier findings on personality and smartphone usage, we hypothesize that extraverts are more active on WhatsApp, because, in general, they are likely to place more value on being connected with people. Of note, the aforementioned study [9] has demonstrated that extraverts have more positive attitudes towards WhatsApp. Although this study did not include directly recorded behaviour, this earlier finding lends support to our hypothesis.

## Methods

### Study design

N = 2,418 participants (1,468 male) were recruited to the present study.^b^ Mean age of participants was 24.64 years (SD = 10.44). In the following also numbers on education in percentages are presented: no education = 148 (6.1%), Volks-/Hauptschulabschluss (streamed secondary school for lesser able students) = 215 (8.9%), secondary school leaving certificate (Mittlere Reife) = 501 (20.7%), vocational baccalaureate diploma (Fachabitur) = 302 (12.5%), A-level (Abitur) = 636 (26.3%), university of applied sciences degree (Fachhochschulabschluss) = 158 (6.5%), and university degree (Hochschulabschluss) = 458 (18.9%). As mass media from Germany reported about our application (see below), by far most of the users of the sample very likely were from Germany. Unfortunately, we did not ask for citizenship.

All participants downloaded our custom developed application called Menthal, which tracked participants’ smartphone use. All participants provided the research team with their anonymized smartphone data profile. For reasons of anonymity, we only collected meta-data of the smartphone. Before downloading the application, all participants provided digital consent to be a participant of our study in exchange for a free service as described in the next section. Ethical approval for the study (including the following described procedure) was obtained from the local ethic committee at the University of Bonn, Germany.

In exchange for providing their data to our research team, participants received insights into their smartphone usage via the custom app. Here, they could see how much time they spent on the smartphone and which applications have been used most often (not only a focus on WhatsApp). This feedback facilitated the recruitment of such a large sample. For convenience, information on personality was completed via a questionnaire integrated into the app. Upon provision of this information, the app provided participants with a short description of their personality profile in the form of graphs.

All participants completed items on age, gender, education and personality. Personality was assessed with a short questionnaire reflecting the Five Factor Model of Personality (Big Five Inventory, BFI, [17]). This questionnaire comprises ten items measuring Extraversion, Neuroticism, Openness to Experience, Agreeableness and Conscientiousness. Each dimension is measured with two items. Thus, each dimension has a score between 2 and 10 for each participant. Items were completed via a five point Likert scale ranging from “disagree strongly” [1] to “agree strongly” [5].

### Tracking the interaction with the smartphone

User interaction was recorded directly on the phone, and the resulting data was forwarded to our servers on a daily basis. We observed around thirty different types of interaction events, e.g. indicating that the phone was unlocked, the user switched from one app to another, or the phone went to “sleep mode”. Some of these events are rather cryptic,^c^ making it difficult to infer the precise nature of the interaction between the user and their smartphone.

We will now introduce the concepts of ‘phone sessions’ and ‘app sessions’. The former indicate a period of uninterrupted interaction with the phone, the latter refers to continuous interaction with an app, e.g. using the WhatsApp messaging service. Regardless of the exact underlying events, app sessions can be represented in a convenient to read format as [user-ID, app-name, start-time, end-time]. It is worth noting that we have only considered visual interaction with the phone. Thus, listening to MP3 music or even talking on the phone while the screen is turned off, does not contribute significantly to either phone or app sessions. Here, we need to add a detail: if you are calling someone the first seconds of calling contributed to phone sessions, but only until the screen went dark.

In order to ensure sufficient data per user, only participants for whom a minimum of 4 weeks’ worth of data were collected were considered in the analysis. In order to reduce any effect of feeling “observed”, we discarded the first week’s data. Due to unforeseeable faults or accidents, data can become faulty or lost, necessitating modest data cleaning processes. For example, if a user engages with WhatsApp and the subsequent “phone off” event is lost, it may appear as if s/he used the messenger for an incredible 8 h (i.e. until the next time s/he uses the phone). We thus excluded data from days for which the user failed to transmit any data, or for which it appeared that phone use exceeded 10 h. The former comprise less than 16.8% of all days under consideration, the latter less than 1.2%. Similarly, we removed all app sessions longer than 6 h. In total, we studied 2,418 participants, of which 2,281 actively used WhatsApp.

## Results

### Age, gender, education and duration of daily general smartphone and WhatsApp Usage

**Fig. 1:**
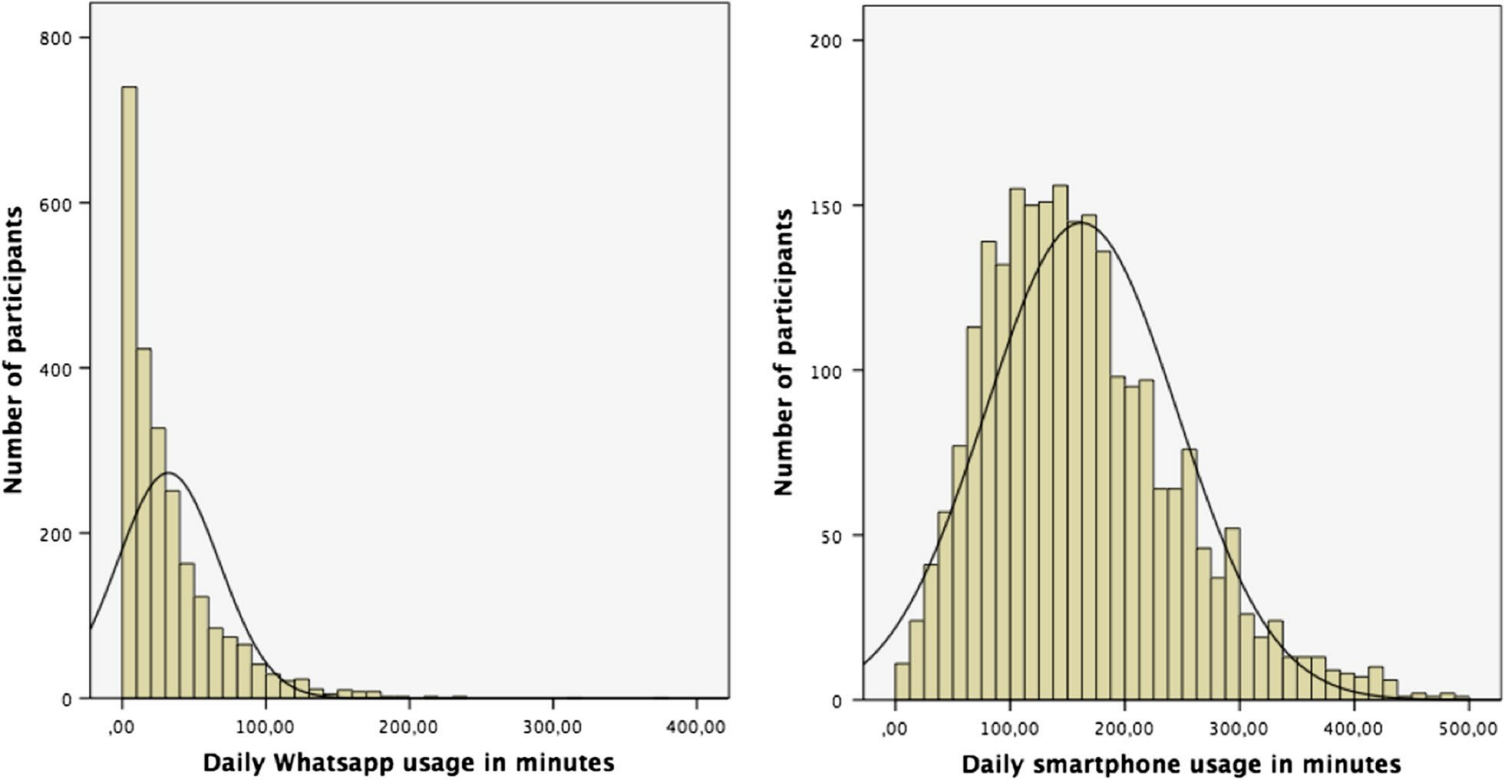
Distribution of participants with respect to WhatsApp usage (*left*) in minutes and daily smartphone usage (*right*).

The mean of typical daily smartphone usage was 161.95 min (SD = 83.36). The mean of typical daily WhatsApp usage was 32.11 min (SD = 35.36). Thus, WhatsApp alone accounted for about 20% (19.83%) of typical daily smartphone use in the present sample. Gender strongly influenced WhatsApp usage (F_(1,2416)_ = 82.35, p < .001), but not general smartphone usage (F_(1,2416)_ = 2.56, p = .11). Of note, non-parametric testing revealed comparable results (U = 514038,00, p < .001) for WhatsApp usage (a non normally distributed variable, as depicted in Fig. 1). Females used WhatsApp for 40.08 min (SD = 36.88) each day. In contrast, males used WhatsApp only for 26.94 min (SD = 33.34) per day. Education was negatively associated with daily smartphone usage (rho = −.20, p < .001), but not significantly associated with WhatsApp usage (rho = −.03, p = .22). Age was not associated with general daily smartphone usage (r = −.01, p = .56), but was inversely linked to daily WhatsApp usage (r = −.33, p < .001; compared with rho = −.41, p < .001). Figure 1 reveals that in contrast to “daily smartphone usage” the variable “daily WhatsApp usage” has a skewed distribution. Thus, we also report statistical results from non-parametric testing for this variable.

Some further notes: In n = 137 participants (5.67%) no WhatsApp activity could be recorded, which can be interpreted to mean that these users have either never installed WhatsApp or that they have installed it, but not use it. Although the group of WhatsApp non-users was very small (thus emphasising the importance of WhatsApp to typical smartphone users), we compared users (mean-daily usage >0) and non-users of WhatsApp on socio-demographic variables and personality. This contrast revealed that WhatsApp users are younger (U = 99604.50, p < .001), more often female (Chi^2^ = 18.41, df = 1, p <  .001), more extraverted (U = 121912.00, p <  .001) and less conscientious (U = 140396.50, p =  .04). Education just fell short of significance between the samples (U = 141425.00, p =  .057, compare with Chi^2^ = 7.72, p = .26), with WhatsApp non-users being slightly more educated. All relevant descriptive statistics are reported in Table 1 (the results from this comparison need to be viewed carefully, because the group of non-WhatsApp users was very small).

**Table 1:** Descriptive statistics on mean-scores of personality, age, education and gender distribution in participants without WhatsApp activity and with WhatsApp activity >0.00 min each day

	Users with no activity on WhatsApp (n = 137)	Users with activity on WhatsApp >0.00 min (n = 2,281)
Age	M = 32.04 (SD = 13.17)	M = 24.20 (SD = 10.09)
Gender	107 males/30 females	1,361 males/920 females
Education	M = 4.67 (SD = 1.90)	M = 4.38 (SD = 1.78)
Extraversion	M = 5.97 (SD = 1.99)	M = 6.74 (SD = 1.96)
Neuroticism	M = 5.57 (SD = 1.98)	M = 5.81 (SD = 1.99)
Conscientiousness	M = 6.64 (SD = 1.80)	M = 6.30 (SD = 1.73)
Agreeableness	M = 6.24 (SD = 1.73)	M = 6.05 (SD = 1.61)
Openness to Experience	M = 7.07 (SD = 1.85)	M = 7.01 (SD = 1.88)

### Personality and duration of daily general smartphone and WhatsApp usage

The duration of daily WhatsApp use is positively associated with Extraversion (rho = .18, p < .001), Neuroticism (rho = .07, p < .001) and inversely associated with Conscientiousness (rho = −.13, p < .001). The remaining correlations with Openness to Experience and Agreeableness showed no significant associations. Given the gender effects in the context of WhatsApp usage, we also provide correlations with males and females separately (see Table 2). The association between Extraversion and WhatsApp usage was double the size in males compared to females (compare rho = .19 vs. rho = .10). The opposite pattern could be observed for Conscientiousness, with higher correlations in females compared to males (compare rho = −.18 vs. rho = −.12). The remaining correlation would not hold for multiple testing correction procedures.

**Table 2:** Correlations between personality and daily WhatsApp usage in minutes for the complete sample and the subsamples consisting of only males and females (all two sided tests)

		Extraversion	Neuroticism	Conscientiousness	Agreeableness	Openness
Total sample n = 2,418	WhatsApp usage	rho = .18, p < .001	rho = .07, p < .001	rho = −.13, p < .001	rho = .001, p = .96	rho = −.01, p = .54
Males n = 1,468	WhatsApp usage	rho = .19, p < .001	rho = .002, p = .94	rho = −.12, p < .001	rho = −.01, p = .77	rho = −.04, p = .09
Females n = 950	WhatsApp usage	rho = .10, p = .002	rho = .04, p = .26	rho = −.18, p < .001	rho = .03, p = .37	rho = −.07, p = .046

## Discussion

The present study provides strong evidence that WhatsApp accounts for a large proportion of typical daily smartphone usage. About 20% of the smartphone behaviour observed in the current study can be accounted for by WhatsApp usage. Females use WhatsApp for longer time periods than males; our data revealed that females use WhatsApp about 13 min longer than males on a daily basis. In addition to gender, age also contributed to WhatsApp usage. Younger participants tend to have longer daily WhatsApp usage. In general, the present data are in line with the literature dealing with the use of social networks. This literature suggests that females also use Facebook more and that younger people tend to be especially active on Facebook [18]. It is worth noting that WhatsApp may not be directly comparable to social network services such as Facebook, as WhatsApp is primarily a communication service. Nevertheless, humans can communicate via both Facebook and WhatsApp, thus some similarities can be observed. In the present study, we focused on WhatsApp, because it accounts for a greater proportion of phone usage compared to the Facebook app (post hoc analyses revealed that Facebook usage showed a mean day level of 15.19 ± 17.98 min (9.38%); far less than that of WhatsApp). See additional information in the Additional file 1.

From a perspective of a personality psychologist, we observed that Extraversion is of high importance in understanding WhatsApp usage. In keeping with earlier smartphone studies on Extraversion and call behaviour (e.g. [19, 20]), extraverts also use WhatsApp for longer durations compared with introverts. This was hypothesized, because extraverts usually reach out more often to their social networks than introverted individuals. Moreover, Sultan [9] suggested that extraverts have more positive attitudes towards WhatsApp compared to introverts. As the positive correlation between WhatsApp use and Neuroticism was very small in the present study, we will not discuss it further. However, a recent study [21] suggested that high neurotics tend to use Facebook more as it facilitates communication without face-to-face interaction; a hypothesis that may warrant further investigation. Finally, Conscientiousness was inversely correlated with WhatsApp usage. This finding fits with other studies showing that conscientious humans handle their digital consumption better and are less prone to Internet addiction (e.g. [22]). Conscientious humans can be described as punctual, and diligently follow their daily routines. No other clear patterns emerged from the present dataset. We acknowledge that the present study represents a descriptive approach to smartphone behaviour. The aim of the study was to provide a record of smartphone demographics for research in psychiatry/psychology and related fields from a large-scale investigation to provide an empirical account of smartphone usage not relying on self-report.

Some further notes: Compared to our earlier study on personality and phone call behaviour, the present correlations are smaller, which probably is due to the more unreliable questionnaire BFI (we used the NEO-FFI in our earlier study, which has better psychometric properties than the short questionnaire used in the present study). This can be also observed when comparing our results on mobile phone behaviour and personality [20] with those from Chittaranjan et al. [16]. We believe that use of the BFI is acceptable due to the large sample size in the present study. Longer personality inventories would likely have resulted in a significantly smaller number of participants. Moreover, the authors of the BFI suggest that “the BFI-10 possesses acceptable psychometric properties” (p. 210, [17]), although the shorter version is accompanied by “substantial losses in comparison to the full-scale BFI”.

A further limitation should be noted: The present study focused on the variable “duration of daily WhatsApp usage in minutes”. For ethical reasons we refrained from the direct analyses of word content in WhatsApp messages. Furthermore, future studies might be interested in considering the number of incoming and outgoing messages, which, from our point of view, could yield interesting additional variables. Although these variables are clearly interesting, we are of the opinion that the variable “duration of daily WhatsApp usage in minutes” investigated in the present study gives the most important overview on WhatsApp usage. Most importantly, the above mentioned associations between socio-demographic variables, personality and WhatsApp usage could not only be observed when investigating all participants, but also in a contrast between participants using WhatsApp and those who are not using WhatsApp.

## Conclusions

The present study is, to our knowledge, one of the first to investigate WhatsApp behaviour. Given the large number of WhatsApp users worldwide and the high daily duration of use (as presented in this paper), it is of great importance to better understand this communication channel. The present study represents a starting point in this endeavour.

## Endnotes

^a^It should be noted that this communication application is not well-known in every country. But similar applications with different names are used around the globe.

^b^Initially, N = 3,272 participants were tracked, but only N = 2,418 are included in the present study as the remaining participants did not complete the information on socio-demographic variables/personality or they filled in questionnaires twice. Users became aware of the app service via mass media coverage. As with many studies in the field, our sample is not representative, but it has the clear advantages of sufficient sample size and directly recorded behaviour.

^c^Many of these make no sense to readers unfamiliar with Android internals. In addition, the same task can usually be represented by a range of different events. Consider the simple task of switching off the phone, which can be conducted by: (a) powering it down, (b) locking the screen, (c) the phone autolocking itself, or (d) the phone slipping in daydream mode. Each of these methods will be represented by a different type of event. In total, this renders log-files of a phone rather unreadable to humans.

## Additional file


**Additional file 1.** In the supplementary material results on usage of the Facebook app will be presented.
