# Supplementary material for: Smartphone usage in the 21st century: who is active on WhatsApp?
Source: BMC Res Notes. 2015 Aug 4;8:331. doi: 10.1186/s13104-015-1280-z (PMC4522968; doi:10.1186/s13104-015-1280-z)
Supplement: Supplementary file 1 — Additional file 1. In the supplementary material results on usage of the Facebook app will be presented. [file 13104_2015_1280_MOESM1_ESM.docx]

*Additional file 1*

Some further notes: In n = 425 participants (17.58%)^[[1]](#footnote-1)^ no Facebook activity could be recorded, which either means that they had not Facebook installed or had Facebook installed and did not use it. For future research endeavors we also want to report the correlations between Facebook app usage and socio-demographic variables, but also with personality traits.

*Age, gender and duration of daily Facebook usage*

Females used Facebook significantly longer than males (F_(1,2416)_=66.14, p < .001; mean = 18.84 (SD=19.68) vs. mean = 12.83 (SD = 16.37)). Non-parametric testing led also to a highly significant result (U = 562658,50, p < .001). Age was inversely associated with Facebook usage (rho = -.18, p < .001; r = -.18, p < .001). No association with education was visible (rho = -.02, p = .39).

*Personality and duration of daily Facebook usage*

The length of daily Facebook usage is positively associated with Extraversion (rho = .08, p < .001) and Neuroticism (rho = .06, p = .005) and inversely with Conscientiousness (rho = -.08, p < .001). After correction for multiple testing, the remaining results with respect to the personality analysis are not significant for the complete sample. Given the gender effects in the context of Facebook usage, we also provide correlations with respect to males and females separately. Here, the negative associations between Conscientiousness and Facebook usage and the same for Openness and Facebook usage in the female sample are noteworthy. These associations are much higher than those observed in males.

Table S1: Correlations between personality and daily Facebook usage on the smartphone in minutes for the complete sample and the subsamples consisting of only males and females (all two sided tests)

|  |  | Extraversion | Neuroticism | Conscientiousness | Agreeableness | Openness |
| --- | --- | --- | --- | --- | --- | --- |
| Total sample n = 2418 | Facebook usage | rho =.08,  p < .001 | rho =.06,  p = .005 | rho =-.08,  p < .001 | rho =.003,  p = .87 | rho =-.05,  p = .03 |
| Males n = 1468 | Facebook usage | rho =.07,  p = .01 | rho = .02,  p = .54 | rho =-.08,  p = .002 | rho =-.03,  p =.34 | rho =-.05,  p = .08 |
| Females n = 950 | Facebook usage | rho =.05,  p = .09 | rho = .02,  p = .59 | rho =-.11,  p = .001 | rho =.05,  p = .09 | rho =-.12,  p < .001 |

1. n = 7 had an activity < 0.005 minutes and were adjusted downwards – hence included in the no activity group of Facebook, the same is true for n = 1 for the WhatsApp analysis [↑](#footnote-ref-1)
